# First identification and coinfection detection of *Enterocytozoon bieneusi, Encephalitozoon* spp., *Cryptosporidium* spp. and *Giardia duodenalis* in diarrheic pigs in Southwest China

**DOI:** 10.1186/s12866-023-03070-x

**Published:** 2023-11-11

**Authors:** Samson Teweldeberhan Ghebremichael, Xianzhi Meng, Yujiao Yang, Amanuel Kidane Andegiorgish, Zongrun Wu, Jie Chen, Junhong Wei, Tian Li, Jialing Bao, Zeyang Zhou, Guoqing Pan

**Affiliations:** 1 State Key Laboratory of Resource Insects, Southwest University, Beibei, Chongqing, 400715 China; 2 Chongqing Key Laboratory of Microsporidia Infection and Control, Southwest University, Beibei, Chongqing, 400715 China; 3 Department of Biology, Mai Nefhi College of Science, Eritrea Institute of Technology, Asmara, Eritrea; 4 College of Veterinary Medicine, Southwest University, Chongqing, China; 5 Epidemiology and Health Statistics, Xi’an Jiaotong University, No.76 Yanta West Road Xi’an, Shaanxi, 710061 China; 6 Chongqing Normal University, Chongqing, 400047 China

**Keywords:** *Enterocytozoon bieneusi*, *Encephalitozoon* spp., Internal transcribed spacer (ITS), *Cryptosporidium* spp., *Giardia duodenalis*, Diarrheic pigs, Coinfection

## Abstract

**Background:**

*Enterocytozoon bieneusi, Encephalitozoon* spp., *Cryptosporidium* spp., and *Giardia duodenalis (G. intestinalis)* are enteric pathogens that cause diarrhea in pigs. This study aimed to determine the prevalence of these enteric parasites and their coinfection with *E. bieneusi* in diarrheic pigs in Southwest China (Chongqing and Sichuan) using nested polymerase chain reaction (nPCR) based methods.

**Results:**

A total of 514 fecal samples were collected from diarrheic pigs from 14 pig farms in Chongqing (five farms) and Sichuan (nine farms) Provinces. The prevalence of *Encephalitozoon* spp., *Cryptosporidium* spp. and *G. duodenalis* was 16.14% (83/514), 0% (0/514), and 8.95% (46/514), respectively. Nested PCR revealed 305 mono-infections of *E. bieneusi*, six of *E. cuniculi*, two of *E. hellem*, and nine of *G. duodenalis* and 106 concurrent infections of *E. bieneusi* with the other enteric pathogens. No infections of *E. intestinalis* and *Cryptosporidium* species were detected. The highest coinfection was detected between *E. bieneusi* and *E. cuniculi* (10.5%, 54/514), followed by *E. bieneusi* and *G. duodenalis* (5.8%, 30/514) and *E. bieneusi* and *E. hellem* (2.9%, 15/514). *E. bieneusi* was the most frequently detected enteric pathogen, followed by *E. cuniculi*, *G. duodenalis* and *E. hellem*. There was a significant age-related difference in the prevalence of *E. cuniculi* in fattening pigs (χ^2^ = 15.266, df = 3, *P* = 0.002) and *G. duodenalis* in suckling pigs (χ^2^ = 11.92, df = 3, *P* = 0.008) compared with the other age groups. Sequence analysis of the ITS region of *Encephalitozoon* species showed two genotypes (II and III) for *E. cuniculi* and one (TURK1B) for *E. hellem*. Only *G. duodenalis* assemblage A was identified in all nested PCR-positive samples. *E. bieneusi* was found more often than other enteric pathogens.

**Conclusions:**

This study showed that *E. bieneusi, Encephalitozoon* spp. [*E. cuniculi* and *E. hellem*] and *G. duodenalis* were common enteric parasites in diarrheic pigs in Chongqing and Sichuan Provinces. In case of both mono-infection and coinfection, *E. bieneusi* was the most common enteric pathogen in diarrheic pigs. Thus, it may be a significant cause of diarrhea in pigs. Precautions should be taken to prevent the spread of these enteric parasites.

**Supplementary Information:**

The online version contains supplementary material available at 10.1186/s12866-023-03070-x.

## Introduction

In pigs, diarrhea is one of the most significant health issues leading to poor productivity and death. Particularly in suckling and weaned pigs, diarrhea results in significant financial losses for the pig industry [1, 2]. Diarrhea disease causes 11.5–29.5% of all pig deaths [3]. It mostly results from a lack of protection of the mother, the environment, and a high infection pressure from enteric pathogens (microsporidia, bacteria, viruses, and parasites) alone or in combination [1, 4]. *Enterocytozoon bieneusi*, *Encephalitozoon* spp., *Giardia duodenalis*, and *Cryptosporidium* spp. are common enteric pathogens that have been found in a wide range of hosts of domestic animals, wild animals and mammals worldwide [5–8].

*Microsporidia* are single-celled obligate intracellular parasites that form spores and are currently thought to be most closely related to the fungal kingdom [9, 10]. They infect many invertebrates and vertebrates, including humans and pigs [11].To date, more than 220 genera and 1,700 species of microsporidia have been identified in different hosts [12]. Among them, 17 species were reported in humans, with *Enterocytozoon bieneusi* and *Encephalitozoon* species as the most common species that infect humans, domestic animals and wild animals and cause almost all gastrointestinal infections [9, 13–15].

*Encephalitozoon cuniculi* is a microsporidian parasite that lives inside cells and produces spores. It can infect numerous mammalian species, including lagomorphs, rodents, dogs, cats, horses, ruminants, wild and exotic carnivores, nonhuman primates, humans, and bird species [16]. *E. cuniculi* was initially detected in pigs in 2007 [17]. This microsporidian species was discovered in one of six samples of swine feces and one of six samples of swine wastewater [17]. According to the number of 5`-GTTT-3` repeats in the ITS sequence of the ribosomal RNA gene, four different genotypes (genotypes I to IV) of *E. cuniculi* can be distinguished [18, 19], and their common names refer to the animal species from which they were first isolated: Genotypes I, II, III, and IV show three-, two-, four-, and five- repeats of the sequence 5`-GTTT-3`, respectively [18, 19]. Genotype I is known as the “rabbit strain,“ Genotype II as the “mouse strain,“ Genotype III as the “dog strain,“ and Genotype IV as the “human strain” [19, 20]. Moreover, ITS sequence analysis revealed that *E. hellem* has four genotypes (1 to 4) [21]. However, the ITS sequence of *E. intestinalis* does not appear to vary within the species [22].

*Giardia duodenalis* and *Cryptosporidium* spp. are common unicellular enteric protozoan parasites and have been found worldwide [23, 24]. They infect a wide variety of vertebrate hosts (such as humans, sheep, pigs, cattle, dogs, and cats), cause diarrhea, and are responsible for many disease outbreaks that spread through water and food in humans and nonhuman animals, particularly those who do not have strong immune systems or are already sick [24, 25]. According to a recent study by Dong et al. [26], the overall prevalence of *Cryptosporidium* worldwide is estimated to be 7.6%, with an average prevalence of 4.3% and 10.4% in developed and developing countries, respectively. However, a prevalence as high as 69.6% has been reported in Mexico [26]. In comparison, giardia infection rates (prevalence) range from 0.4 - 7.5% and 8–30% in developed and developing countries, respectively [27]. Humans can obtain *Cryptosporidium* oocysts and *Giardia* cysts directly or indirectly through contaminated water, food, and pastures [23]. The Food and Agriculture Organization of the United Nations (FAO) ranked *Cryptosporidium* spp. and *G. duodenalis* fifth and eleventh, respectively, on a list of 24 parasites that can be spread through food [23]. To date, at least 45 *Cryptosporidium* spp. and more than 120 genotypes have been identified; among these 19 species, four genotypes were found in humans. *C. hominis* and *C. parvum* are the most common species that cause cryptosporidiosis in humans [24, 25]. Currently, there are eight confirmed *G. duodenalis* assemblages (A- H) [24]; of these assemblages, A and B commonly infect humans and animals, while the remaining six (C-H) are host-specific [28].

Pigs have been reported to serve as hosts for different zoonotic species of those mentioned above that are enteropathogenic and are considered a possible source of human infections [7, 8]. In our previous work, we addressed the prevalence and genotypic distribution of *E. bieneusi* [29]. However, there is not much information about the coinfection of *E. bieneusi* with other enteric pathogens in diarrheic pigs worldwide. In addition, pigs are a common food source in different parts of the world. Whether a pig is a typical host for enteric pathogens and thus poses human health threats is not yet understood. Therefore, the present study aimed to determine the coinfection of *E. bieneusi* and the prevalence of enteric pathogens in diarrheic pigs, with special emphasis on *Encephalitozoon* species, *G. duodenalis* and *Cryptosporidium* species, in Chongqing and Sichuan Provinces from September 2021 to March 2022.

## Methods

### Sample collection and DNA extraction

A total of 514 fresh fecal samples were collected from 14 large-scale pig farms in Chongqing (five) and Sichuan (nine) Provinces from September 2021 to March 2022. All fecal specimens were collected directly from the middle part of the feces on the ground after defecation using sterile disposable gloves and placed in individual plastic containers. The pig breeds used in this study were Pig Improvement Company (PIC) pigs. Each pig’s collection date, age and identification number were recorded at the time of sampling. The fecal samples of pigs were grouped into four age groups (Table 1). All pigs were in a diarrheic condition during the time of sampling. The piglets were dewormed twice, at 40–50 and 120 days, using albendazole. Sows were dewormed once at approximately four months. The albendazole dosages used for piglets and sows were in the range of 0.07 ~ 0.1 g per kg of body weight. All samples were transported immediately to the laboratory on-ice packs and kept at -20 ℃ until DNA extraction.

**Table 1:** Primers and PCR conditions for identifying *Encephalitozoon* spp. by targeting the ITS region, *Cryptosporidium* spp. by targeting the SSU rRNA gene, and *G. duodenalis* by targeting the bg gene

Organisms	Gene	Primer name	Primer sequence(5’-3’)	Amplicon size (bp)	Reaction condition	Reference
*Encephalitozoon* spp.	*ITS*	MSP-1	TGAATG(G/T)GTCCCTGT	~ 300	94 °C for 3 min; 35 cycles of 94 °C for 45 s, 58 °C for 45 s, and 72 °C for 1 min; 72 °C for 7 min	[31]
		MSP-2 A	TCACTCGCCGCTACT			
		MSP-3	GGAATTCACACCGCCCGTC(A/G)(C/T)TAT			
		MSP-4 A	CCAAGCTTATGCTTAAGT(C/T)(A/C)AA(A/G)GGGT			
*Cryptosporidium* spp.	*SSU rRNA*	F1	TTCTAGAGCTAATACATGCG	830	94 °C for 3 min; 35 cycles of 94 °C for 45 s, 55 °C for 45 s, and 72 °C for 1 min; 72 °C for 7 min	[33, 34]
		R1	CCCATTTCCTTCGAAACAGGA			
		F2	GGAAGGGTTGTATTTATTAGATAAAG			
		R2	AAGGAGTAAGGAACAACCTCCA			
*Giardia duodenalis*	*bg*	bg-F1	AAGCCCGACGACCTCACCCGCAGTGC	511	95 °C for 15 min; 35 cycles of 95 °C for 30 s, 55 °C for 30 s, and 72 °C for 1 min; 72 °C for 7 min	[32]
		bg-R1	GAGGCCGCCCTGGATCTTCGAGACGAC			
		bg-F2	GAACGAGATCGAGGTCCG			
		bg-R2	CTCGACGAGCTTCGTGTT			
*E. bieneusi*	*ITS*	EBITS3	GGTCATAGGGATGAAGAG	390	94 °C for 3 min; 35 cycles of 94 °C for 30 s, 57 °C (55 °C for 2nd PCR) for 30 s, and 72 °C for 40 s; 72 °C for 10 min	[35]
		EBTIS4	TTCGAGTTCTTTCGCGCTC			
		EBITS1	GCTCTGAATATCTATGGCT			
		EBITS2.4	ATCGCCGACGGATCCAAGTG			

All the specimens were washed twice using distilled water to remove any preservatives or impurities and sieved through an 8 cm diameter sieve with a pore size of 45 μm. Then the filtrates were concentrated by centrifugation at 15,000 xg for 10 min. Genomic DNA was extracted from approximately 200 µL (200 mg) of each processed sample using an E.Z.N.A.® Mag- Bind Stool DNA Kit (OMEGA, Biotek Inc., Norcross, GA, USA), as recommended by the manufacturers. DNA was eluted in 50 µL of double-deionized distilled water, and DNA quantification was carried out using a DeNovix DS-11 + spectrophotometer/fluorometer (DeNovix, Wilmington, United States) instrument. Finally, the extracted DNA was kept at -20 ℃ in a freezer until PCR analysis was performed.

### PCR amplification

*Encephalitozoon* species were detected by nested PCR amplification of the fragment (approximately 305 bp) of the internal transcribed spacer (ITS) as previously described [30, 31]. In brief, the ITS was amplified by MSP-1 and MSP-2 A and by MSP-3 and MSP-4 A as outer- and inner primer pairs, respectively. The outer primer pair amplifies a large region containing the SSU, ITS, and LSU rRNA genes in several species of microsporidians. In contrast, the inner primer pair amplifies sequences in only *Encephalitozoon* spp. (*E. cuniculi*, *E. intestinalis* and *E. hellem*) [10].

*G. duodenalis* was screened using nested PCR amplification of the fragment (~ 500 bp) of the β-giardin (bg) gene as described previously [32]. *Cryptosporidium* spp. were screened using nested PCR amplification of the segment (~ 830 bp) of the small subunit rRNA (SSU rRNA) gene with previously published primers and PCR cycle settings [33, 34].

All PCRs were carried out in 25 µL reaction mixtures, including 12.5 µL of rTaq PCR Master Mix (Sanger Biotech Co., Ltd., Shanghai, China), 0.5 µL of each primer (0.4 mM), 1 µL of each DNA sample, and 10.5 µL of double distilled water. Positive and negative controls were used in all the PCR tests performed. Finally, the secondary PCR products were subjected to electrophoresis on a 1.5% agarose gel and visualized by the ChemiDoc XRS + Gel Imaging System (Bio-Rad, California, United States). Moreover, separate workplaces were employed for DNA extraction, PCR preparation and amplification to avoid contamination. The primers and PCR conditions are summarized in Table 2.

**Table 2:** Prevalence of enteric pathogens in pigs with diarrhea by age

Age groups	No. tested	E. bieneusi*	*E. cuniculi*	*E. hellem*	*G. duodenalis*	*Cryptosporidium* spp.
		No. of Positive (%)	No. of positive (%)	No. of positive (%)	No. of positive (%)	No. of positive (%)
Suckling piglets (Age ≤ 1 month)	326	262 (80.4)	29 (8.9)	15 (4.6)	39 (10.8)	0 (0.0)
Weaned pigs (1 to 3 months)	17	11 (64.7)	0 (0.0)	0 (0.0)	0 (0.0)	0 (0.0)
Fattening pigs (4 to 6 months)	65	47 (72.3)	15 (23.1)	1 (1.5)	5 (7.7)	0 (0.0)
Sow (≥ 6 months)	106	90 (84.9)	18 (17.0)	5 (4.7)	2 (2.0)	0 (0.0)
**Total**	**514**	**410 (79.8)**	**62 (12.0)**	**21 (4.0)**	**46 (8.9)**	**0 (0.0)**

E. bieneusi^*^= data from Ghebremichael, Meng [29]

### Nucleotide sequencing and analysis

All the samples that produced a positive result with nested PCR amplicons of targeted genes were directly sent for bidirectional sequencing to Sangon Biotech Co., Ltd. (Shanghai, China). The nucleotide sequences obtained in this study were edited using Snap-Gene version 5.1 sequence analysis software (TechnelysiumPty Ltd., South Brisbane, Australia). The Basic Local Alignment Search Tool (BLAST) searches were performed to determine whether there were any similarities with the sequences already deposited in GenBank. Then, ClustalX 2.1 [http://www.clustal.org] was used to identify the *Encephalitozoon* species and genotypes by comparison with reference sequences of *Encephalitozoon* spp. and genotypes that were downloaded from the National Center for Biotechnology Information [https://www.ncbi.nlm.nih.gov/]. The same procedures were followed to determine the species and subtypes of *Cryptosporidium* and *G. duodenalis* assemblages. Representative *E. cuniculi*, *E. hellem* and *G. duodenalis* nucleotide sequences found in diarrheic pigs have been deposited in GenBank at the National Center for Biotechnology Information with the accession numbers OR058746 to OR058756 for *E. cuniculi*, OR058757 for *E. hellem*, and OR091265 to OR091271 for *G. duodenalis*.

### Statistical analysis

Statistical Package for the Social Sciences (SPSS) 22.0 (for Windows, Version, IBM Armonk Corp., New York, NY, USA) was used to compare the prevalence of *Encephalitozoon* spp., *Cryptosporidium* spp. and *G. duodenalis* in diarrheic pigs using the χ2 test. Significance was defined at p < 0.05.

## Results

### Prevalence of enteric pathogens in diarrheic pigs by age group in chongqing and Sichuan Provinces (Southwestern China)

**Fig. 1:**
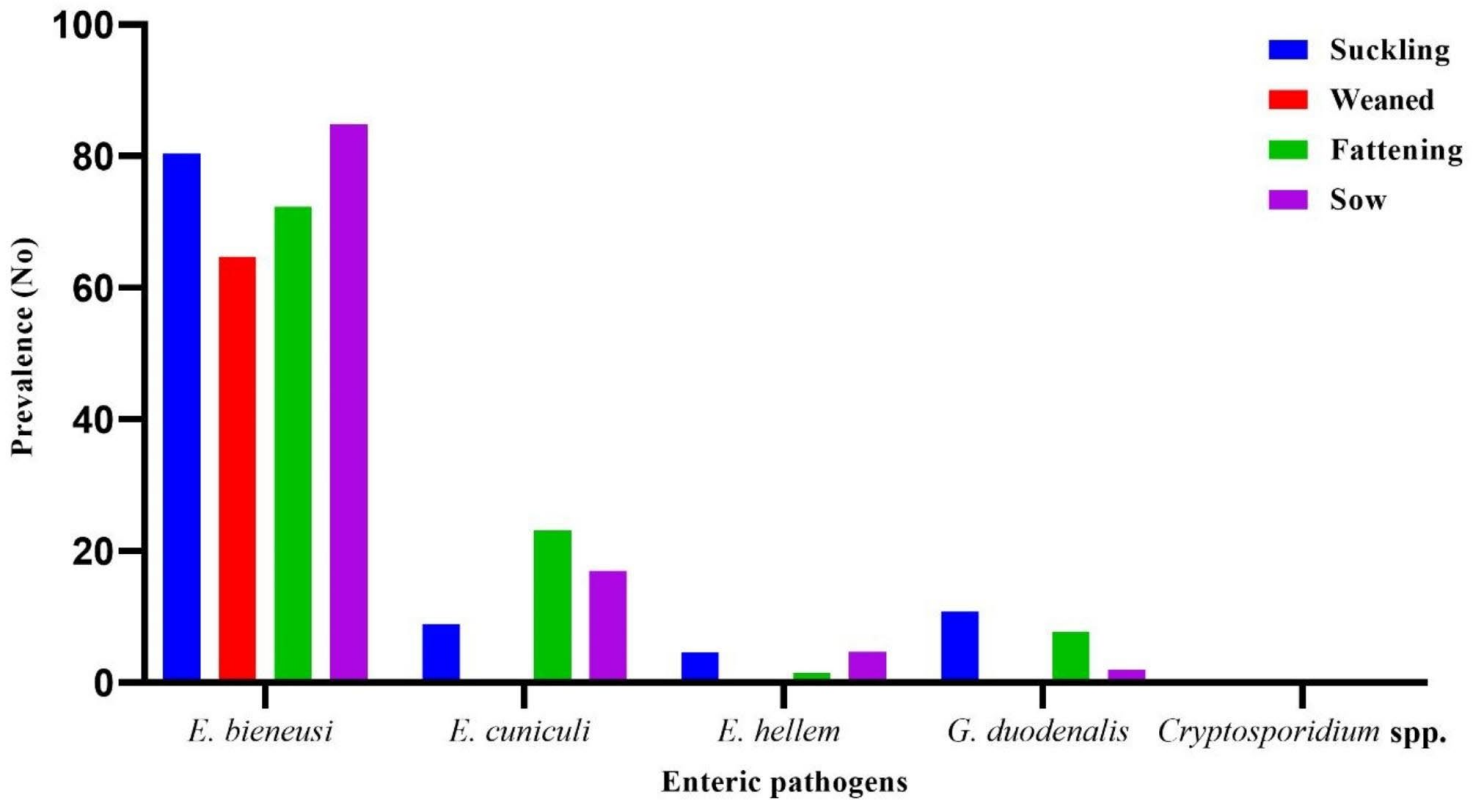
Prevalence of *E. bieneusi*, *E. cuniculi*, *E. hellem*, *G. duodenalis* and *Cryptosporidium* spp. in age groups of diarrheic pigs from Chongqing and Sichuan Provinces

The prevalence of each enteric pathogen in diarrheic pigs in different age groups is described in Table 1. We detected a higher prevalence of *E. bieneusi* infections than of other infections in all age groups (Table 1/Fig. 1) [29]. According to the age distribution, the highest infection rate of *E. cuniculi* was recorded in fattening pigs (23.1%, 15/65), followed by sows (17%, 18/106) and suckling piglets (8.9%, 29/326). In *E. hellem*, the same prevalence (4.7%) was recorded for suckling piglets and sows. No prevalence was found in weaned pigs for the *Encephalitozoon* species (Table 1/Fig. 1). The highest rate of *G. duodenalis* infection was found in suckling (10.8%, or 39/362), followed by fattening pigs (7.7%, or 5/65) and sows (2%, or 2/106), and there were no infections in the weaned age group (Fig. 1).

### Microsporidian detection in pigs

The overall prevalence of microsporidian species in diarrheic pigs was 96.7% (493/510). The prevalence of *E. bieneusi*, *E. cuniculi*, and *E. hellem* were 79.8% (410/514), 12.0% (62/514) and 4% (21/514), respectively (Table 3). No *E. intestinalis* species were detected in the present study. *Encephalitozoon* spp. were found in all samples collected from Chongqing and Sichuan Provinces except for those from farms 8 and 12. The prevalence of *Encephalitozoon* spp. was highest (69.9%) and lowest (0%) in Sichuan suckling pigs on farms 5 and 8, respectively. The infection rate of *Encephalitozoon* species for the sows was highest (60.0%) and lowest (0.0%) on farms 2 and 8, respectively. In Chongqing, 14.3% (2/14) and 0% (0/1) were recorded as the highest and lowest prevalence rates of *Encephalitozoon* spp. in sows from farms 10 and 12, respectively. In fattening pigs, 7 of 25 (28.0%) and 4 of 20 (20.0%) were recorded as the highest and lowest prevalences of *Encephalitozoon* spp. on farms 13 and 10, respectively. In suckling pigs, 2 of 5 (40.0%) and 0 of 6 (0%) were the highest and lowest prevalences recorded on farms 14 and 12, respectively, for *Encephalitozoon* species. The prevalence of *E. cuniculi* in fattening pigs was significantly higher, 23.1% (15/65), than in sows 17% (18/106) (χ^2^ = 15.266, df = 3, P = 0.002). However, there were no statistically significant differences in the prevalence of *E. bieneusi*, and *E. hellem*, even though there was a difference in prevalence among the age groups (χ^2^ = 6.437, df = 3, *P* = 0.092; χ^2^ = 2.129, df = 3, *P* = 0.546) (Table 1]. Genotypes II and III of *E. cuniculi* were found at least once on almost all farms except farms 8, 9, and 12. No *E. hellem* were found on six farms [6, 7, 8, 11, 12 and 14] (Table 3).

**Table 3:** Prevalence and genotypes of *Encephalitozoon* spp. in diarrheic pigs in Chongqing and Sichuan Provinces

Provinces	Farms	№ tested	Eb^*^	Enc. spp.	Mono infection of Ec.	Mixed infection of Eb & Ec.		Mono infection of *E. hellem*	Mixed infection of Eb & Eh	
			No. (%)	No. (%)	Genotype (No.)	No. (%)	Genotype (No.)	Genotype (No.)	No. (%)	Genotype (No.)
Sichuan	1	23	23 (100.0)	5 (21.7)	0	2	PigCE07.01 + III (1), PigCE02.03 + III (1)	0	3	PigCE01.01 + TURK1B (1), PigCE02.01 + TURK1B (1), PigCE08.03 + TURK1B (1)
	2	48	45 (93.8)	10 (20.8)	0	5	B + III (3), PigCE06.02 + III (1), PigCE08.05 + III (1)	0	5	PigCE01.02 + TURK1B (2), PigCE08.02 + TURK1B (1), PigCE07.05 + TURK1B (1), PigCE06.04 + TURK1B (1)
		5	5 (100.0)	3 (60)	0	3	PigCE07.07 + II (1), PigCE07.05 + II (1), PigCE08.05 + III (1)	0		0
	3	50	39 (78.0)	7 (14)	III (1)	3	PigCE08.07 + III (2), PigCE08.11 + III (1)	TURK1B (1)	2	B + TURK1B (1), PigCE08.02 + TURK1B (1)
	4	50	46 (92.0)	16 (32)	0	12	PigCE08.07 + II (1), PigCE07.05 + III (1), PigCE01.04 + III (1), PigCE08.09 + III (1), PigCE08.15 + III (2), PigCE01.06 + III (1), B + III (1), PigCE08.02 + III (3), PigCE08.05 + III (1)		4	PigCE06.01 + TURK1B (1), PigCE07.08 + TURK1B (1), PigCE07.09 + TURK1B (1), B + TURK1B (1)
	5	23	22 (95.7)	16 (69.6)	III (1)	13	PigCE08.07 + III (1), PigCE01.0 2 + III (1), PigCE08.17 + III (1), PigCE08.02 + III (6), PigCE07.01 + III (1), PigCE07.09 + III (2), B + III (1)		2	PigCE08.07 + TURK1B (1), PigCE08.04 + TURK1B (1)
	6	30	28 (93.3)	0 (0)	0		0	0		
		20	13 (65.0)	1 (5)		1	B + III (1)	0		
	7	40	24 (60.0)	0 (0)	0		0	0		
		11	7 (63.6)	1 (9.1.)		1	PigCE08.02 + III (1)	0		
	8	45	34 (75.6)	0 (0)	0		0	0		
		5	5 (100.0)	0 (0)	0		0	0		
	9	50	35 (70.0)	2 (4)	0		0	0	2	PigCE08.02 + TURK1B (1), PigCE08.10 + TURK1B (1)
	**Sub total**	**400**	**326** (81.5)	**61** (15.3)	**III (2)**	**40**	**II (3), III (37)**	**1**	**18**	
Chongqing	10	20	15 (75.0)	4 (20)	0	4	B + II (1), B + III (2), PigCE02.06 + III (1)	0		
		14	14 (100.0)	2 (14.3)	0	1	PigCE01.01 + III (1)		1	PigCE07.01 + TURK1B (1)
	11	20	11 (55.0)	5 (25)	III (2)	3	PigCE08.02 + II (1), PigCE07.02 + II (1), PigCE08.02 + III (1)	0		
	12	9	5 (55.6)	0 (0)	0	0	0	0		
		6	5 (83.3)	0 (0)	0	0	0	0		
		1	0 (0.0)	0 (0)	0	0	0	0		
	13	25	21 (84.0)	7 (28)	0	6	F + II (2), E + III (1), F + III (1), Peru8 + III (1), PigCE05.01 + III (1)	TURK1B (1)	0	
		8	6 (75.0)	0 (0)	0	0	0	0	0	
		6	5 (83.3)	2 (33.3)	0	2	B + III (1), PigCE08.02 + III (1)	0	0	
	14	5	2 (40.0)	2 (40)	II (1), III (1)	0	0	0	0	
	**Sub total**	**114**	**84 (73.7)**	**22 (19.3)**	**II (1), III (3)**	**16**	**II (5), III (11)**	**1**	**1**	
	**Total**	**514**	**410 (79.8)**	**83** **(16.1)**	**II (1), III (5)**	**56 (10.9)**	PigCE07.02 + II (1); PigCE07.05 + II (1); PigCE07.07 + II (1); PigCE08.02 + II (1); PigCE08.07 + II (1); B + II (1); F + II (2); PigCE01.01 + III (1); PigCE01.02 + III (1); PigCE01.04 + III (1); PigCE01.06 + III (1); PigCE02.03 + III (1); PigCE02.06 + III (1); PigCE05.01 + III (1); PigCE06.02 + III (1); PigCE07.01 + III (2); PigCE07.05 + III (1); PigCE07.09 + III (2); PigCE08.02 + III(12);PigCE08.05 + III (3); PigCE08.07 + III (3); PigCE08.09 + III (1); PigCE08.11 + III (1); PigCE08.15 + III (2); PigCE08.17 + III (1); B + III (9); E + III (1); F + III(1), Peru8 + III (1)	TURK1B (2)	19 (3.7)	B + TURK1B (2); PigCE01.01 + TURK1B (1); PigCE01.02 + TURK1B (2); PigCE02.01 + TURK1B (1); PigCE06.01 + TURK1B (1); PigCE06.04 + TURK1B (1); PigCE07.01 + TURK1B (1); PigCE07.05 + TURK1B (1); PigCE07.08 + TURK1B (1); PigCE07.09 + TURK1B (1); PigCE08.02 + TURK1B (3); PigCE08.03 + TURK1B (1); PigCE08.04 + TURK1B (1); PigCE08.07 + TURK1B (1); PigCE08.10 + TURK1B (1)
**Total**					**62/514 (12%)**			**21/514 (4%)**		

Eb = *E. bieneusi*; Enc. spp.= *Encephalitozoon species*; Ec. = *E. cuniculi*; Eb^*^= data from [29]

### Genotype distribution of *Encephalitozoon* spp.

Sequence analysis of the ITS region of *E. cuniculi* revealed two distinct genotypes (II and III), and only one genotype (TURK1B) was identified in *E. hellem* in this study. A total of 83 *Encephalitozoon* spp.-positive samples were amplified and sequenced successfully in diarrheic pigs. Furthermore, most farms harbored at least one *Encephalitozoon* spp.genotype, except farms 8, 9 and 12. For *E. cuniculi*, nine cases of genotype II and fifty-three cases of genotype III were identified. In the present investigation, genotype III was the most prevalent genotype. In both provinces, only genotype TURK1B of *E. hellem* was found (Table 3).

In this study, the sequences of 48 positive samples isolated from diarrheic pigs with genotype III of *E. cuniculi* were 100% identical to the sequence (KX189630) from *Apodemus agrarius*, a striped field mouse in a study from Poland [36]. At the same time, four samples with genotype III sequences were identical and had the highest degree of similarity (99.5–98.97%) with the *Apodemus agrarius* genotype III (KX189630). A genotype III sequence found in one diarrheic pig sample in this study had 99.65% homology to a genotype III sequence (KJ577583) isolate from *Lagurus lagurus* (steppe lemming) in the Czech Republic [37] (Table 4, Figure S1(B)). In addition, eight positive *E. cuniculi* genotype II samples showed complete sequence identity with genotype II (accession No. KX189632) from yellow-necked mouse [*Apodemus flavicollis*] in Poland [36]. One sample of *E. cuniculi* genotype II had 98.92% homology with isolate from yellow-necked mouse (accession No. KX189632) (Table 4, Figure S1(A)). All 21 positive samples of *E. hellem* showed complete sequence similarity to a positive sample of genotype TURK1B (accession No. MT478448) from a budgerigar [*Melopsittacus undulatus*] in Turkey [38] (Figure S1(C)). 

**Table 4:** *E. cuniculi* genotypes determined on the basis of the small subunit rRNA (rRNA) gene locus

Genotype	Accession no.	Nucleotide position								
		130	158	159						
**Genotype II**	KX189632 (Ref. seq.)	**T**	**G**	**G**						
	OR058746_PigCE01-II	T	G	G						
	OR058747_PigCE02-II	C	G	G						
	OR058748_PigCE03-II	T	T	T						
		**71**	**86**	**94**	**135**	**150**	**155**	**159**	**197**	**201**
**Genotype III**	KJ577583 (Ref. seq.)	**T**	**A**	**A**	**T**	**G**	**T**	**T**	**G**	**T**
	OR058749_PigCE04-III	T	A	A	T	G	C	T	G	**C**
	OR058750_PigCE05-III	T	A	A	T	A	T	T	G	**T**
	OR058751_PigCE06-III	T	A	A	A	G	T	C	G	**T**
	OR058752_PigCE07-III	C	A	A	T	G	T	T	G	**T**
	OR058753_PigCE08-III	T	A	T	T	G	T	T	G	**T**
	OR058754_PigCE09-III	T	G	A	T	G	T	T	G	**T**
	OR058755_PigCE10-III	T	A	A	T	G	T	T	A	**T**
	OR058756_PigCE11-III	T	A	A	T	G	T	T	G	**T**

### Prevalence of ***Cryptosporidium*** spp. and ***G. duodenalis***

Among the 514 fecal samples collected from pigs, the prevalence of *G. duodenalis* was 8.95% (46/514), and *Cryptosporidium* spp. were not detected (0%, 0/514). This study identified higher infections (10.0%, 41/410) and lower infections (4.4%, 5/114) of *G. duodenalis* the Sichuan and Chongqing Provinces, respectively. The prevalence of *G. duodenalis* on different farms ranged from 0 to 36%. Among the farms, farm 9 had the highest prevalence (36%, 18/50), followed by farm 5 (17.4%, 4/23) and farm 10 (15%, 3/20), while farms 12, 13, and 14 had no *G. duodenalis* (Table 5). The highest rate of *G. duodenalis* infection was found in suckling pigs (10.8%, 39/326), followed by fattening pigs (7.7%, 5/65) and sows (2.0%, 2/106), and no infections were found in weaned pigs. *G. duodenalis* infection rates varied significantly between the age groups (χ^2^ = 11.92, df = 3, *P* = 0.008) (Table 1).

**Table 5:** Farm-specific prevalence and genetic characterizations of *G. duodenalis* and *Cryptosporidium* spp. in diarrheic pigs in Chongqing and Sichuan Provinces, China

Provinces	Farms	№ tested	*G. duodenalis*					*Cryptosporidium spp.*
			Overall infection		Mono infection	Mixed infection of Eb *& G. duodenalis*		
			No. (%)	Assemblage (No.)	No. (%)	No. (%)	Genotype (No.)	No. (%)
Sichuan	1	23	1(4.4%)	A (1)	0	1	PigCE07.02 + A (1)	0
	2	48	7 (14.6%)	A (7)	0	7	PigCE01.01 + A (2), PigCE01.02 + A (1), PigCE01.03 + A (1), PigCE08.02 + A (1), PigCE07.05 + A (1), B + A (1)	0
		5	0	0	0	0	0	0
	3	50	5 (10.0%)	A (5)	0	5	PigCE06.01 + A (1), PigCE08.02 + A (1), PigCE08.09 + A (1), PigCE08.10 + A (1), PigCE08.12 + A (1),	0
	4	50	0	0	0	0	0	0
	5	23	4 (17.4%)	A (4)	0	4	PigCE01.02 + A (1), PigCE08.07 + A (1), PigCE08.16 + A (1), B + A (1)	0
	6	30	0	0	0	0	0	0
		20	2 (10.0%)	A (1)	1	1	B + A (1)	0
	7	40	2 (5.0%)	A (2)	1	1	B + A (1)	0
		11	0		0	0	0	0
	8	45	2 (4.4%)	A (2)	0	2	PigCE08.02 + A (1), PigCE08.07 + A (1)	0
		5	0	0	0	0	0	0
	9	50	18 (36.0%)	A (18)	6	12	PigCE02.01 + A (1), PigCE07.01 + A (1), PigCE08.02 + A (6), PigCE08.07 + A (2), PigCE08.10 + A (1), B + A (1),	0
	**Subtotal**	**400**	**41** (10.3%)	**A (41)**	**8 (2)**	**33** **(8.3)**	B + A (4), PigCE01.01 + A (2), PigCE01.02 + A (2), PigCE01.03 + A (1), PigCE02.01 + A (1), PigCE06.01 + A (1), PigCE07.01 + A (1), PigCE07.02 + A (1), PigCE07.05 + A (1), PigCE08.02 + A (9), PigCE08.07 + A (4), PigCE08.09 + A (1), PigCE08.10 + A (2), PigCE08.12 + A (1), PigCE08.16 + A (1)	**0**
Chongqing	10	20	**3** (15.0%)	A (3)	1	2	PigCE07.04 + A (1), PigCE08.03 + A (1)	**0**
		14	0	0	0	0	0	0
	11	20	2 (10.0%)	A (2)	0	1	B + A (1), III + A (1)	0
	12	9	0	0	0	0	0	0
		6	0	0	0	0	0	0
		1	0	0	0	0	0	0
	13	25	0	0	0	0	0	0
		8	0	0	0	0	0	0
		6	0	0	0	0	0	0
	14	5	0	0	0	0		0
	**Subtotal**	**114**	**5** (4.4%)	**A (5)**	2	3	B + A (1), PigCE07.04 + A (1), PigCE08.03 + A (1),	**0**
	**Total**	**514**	**46** (8.95%)	**A (46)**	**9 (1.75)**	**37** **(7.2)**	B + A (5), PigCE01.01 + A (2) PigCE01.02 + A (2), PigCE01.03 + A (1), PigCE02.01 + A (1), PigCE06.01 + A (1), PigCE07.01 + A (1), PigCE07.02 + A (1), PigCE07.04 + A (1), PigCE07.05 + A (1), PigCE08.02 + A (9), PigCE08.03 + A (1), PigCE08.07 + A (4), PigCE08.09 + A (1), PigCE08.10 + A (2), PigCE08.12 + A (1), PigCE08.16 + A (1), III + A (1)	**0**

### Distribution of ***G. duodenalis*** assemblages and ***Cryptosporidium*** spp. genotypes

For isolates of *G. duodenalis*, sequence analysis of the β-giardin (bg) gene revealed that all the positive samples belonged to *G. duodenalis* assemblage A (n = 46). Thirty-seven assemblage A sequences shared complete sequence identity with human-, sheep- and cat-derived assemblage A isolated (KP687765, KR075937, KJ027408) from Canada and China, respectively (Table 6). Six assemblage A sequences showed identities of 99.39 to 99.8%, the same as two assemblage A isolates (KP687765, KR075937) derived from humans and sheep in Canada and China, respectively [39, 40] (Table 6, Figure S1(D)). Another three assemblage A sequences were 99.8% identical to a human-derived assemblage A (KP687765) sequence from Canada and a cat-derived isolate (KJ027408) sequence from China.

**Table 6:** *G. duodenalis* assemblage A subtypes defined on the basis of the beta-giardin (bg) gene locus

Accession no.	Nucleotide position							
	83	99	330	358	362	409	453	470
KR075937 (Ref. seq.)	**A**	**G**	**A**	**A**	**A**	**C**	**G**	**A**
OR091265 _PigCE01_Gbg	A	G	A	A	A	C	G	A
OR091266 _PigCE02_Gbg	G	G	A	A	A	C	G	A
OR091267 _PigCE03_Gbg.	A	G	A	A	A	T	G	A
OR091268 _PigCE04_Gbg	A	G	A	A	A	C	A	A
OR091269 _PigCE05_Gbg	A	A	G	A	G	C	G	A
OR091270 _PigCE06_Gbg	A	G	A	G	A	C	G	A
OR091271_PigCE07_Gbg	A	G	A	A	A	C	G	T

### Prevalence of single infection and coinfection of ***E. bieneusi***, ***Encephalitozoon*** spp. [***E. cuniculi*** and ***E. hellem***], ***Cryptosporidium*** spp. and ***G. duodenalis*** in diarrheic pigs in Southwestern China

Table 7 displays the occurrence of single- and mixed infections of enteric pathogens detected in diarrheic pigs. The nested PCR revealed 305 *E. bieneusi* mono-infections, six *E. cuniculi* mono-infections, two *E. hellem* mono-infections, and nine *G. duodenalis* mono-infections (Table 7). However, no infections with *E. intestinalis* and *Cryptosporidium* spp. were detected. Most of the diarrheic pigs that tested positive, 62.6% (322/514), had only one pathogen, and 20.6% (106/514) tested positive for two or three pathogens. Regarding mono-infection, *E. bieneusi* was the most commonly found agent, followed by *G. duodenalis*, *E. cuniculi*, and *E. hellem*.

**Table 7:** Single and mixed infections of *E. bieneusi*, *Encephalitozoon* spp. [*E. cuniculi* and *E. hellem*], *Cryptosporidium* spp. and *G. duodenalis* in diarrheic pigs (total samples n = 514)

Enteric pathogens	No. positive	%
*Enterocytozoon bieneusi*	305	59.3
*Encephalitozoon cuniculi*	6	1.2
*Encephalitozoon hellem*	2	0.4
*Cryptosporidium* spp.	0	0.0
*G. duodenalis*	9	1.8
**Total with single infection**	**322**	**62.6**
*E. bieneusi* + *E. cuniculi*	54	10.5
*E. bieneusi* + *E. hellem*	15	2.9
*E. bieneusi* + *Cryptosporidium* spp.	0	0.0
*E. bieneusi* + *G. duodenalis*	30	5.8
*E. bieneusi* + *E. cuniculi* + *G. duodenalis*	2	0.4
*E. bieneusi* + *E. hellem* + *G. duodenalis*	4	0.8
*E. bieneusi* + *E. hellem* + *Cryptosporidium* spp.	0	0.0
*E. cuniculi +* G. *duodenalis*	1	0.2
**Total with mixed infection**	**106**	**20.6**
**Negative**	**86**	**16.7**

The present study revealed eight and forty-eight positive isolates as coinfections between Genotypes II and III of *E. cuniculi* and *E. bieneusi*, respectively. The highest mixed infection rate was recorded for *E. bieneusi* and Genotype III of *E. cuniculi* with 12 positive isolates of Genotype PigCE08.02, followed by genotypes PigCE08.05 and PigCE08.07, each with three positive isolates, whereas PigCE07.01, PigCE07.09, and PigCE08.15 each had two positive isolates. Two positive isolates had *E. bieneusi* Genotype F and *E. cuniculi* Genotype II. All the remaining genotypes of *E. bieneusi* and *E. cuniculi* genotypes II and III had only one positive isolate each (Table 3). Between *E. bieneusi* and *G. duodenalis* assemblage A, the highest mixed infection rate was recorded for PigCE08.02 with nine positive isolates, followed by Genotype B in five positive isolates, PigCE08.07 with four positive isolates, and PigCE08.01 and PigCE08.10, each with two positive isolates. The remaining positive isolates had one genotype each of *E. bieneusi* and *G. duodenalis* (Table 5).

In total, the highest mixed infection rate was detected between *E. bieneusi* and *E. cuniculi* (10.5%, 54/514), followed by *E. bieneusi* and *G. duodenalis* (5.8%, 30/514) and *E. bieneusi* and *E. hellem* (2.9%, 15/514). *E. bieneusi* was the most frequently detected enteric pathogen, followed by *E. cuniculi*, *G. duodenalis* and *E. hellem*.

## Discussion

Pork is one of the most widely consumed meats in China, and its quality and demand are rising. Pigs are considered one of the most important reservoirs for enteric pathogens (*E. bieneusi*, *E. cuniculi*, *E. hellem Cryptosporidium*, and *G. duodenalis*) [41]. An epidemiological investigation is one of the best ways to learn about the molecular characteristics and diversity of enteric pathogens. Therefore, we examined enteric pathogen prevalence and mixed infection in diarrheic pigs from Chongqing and Sichuan Provinces in Southwestern China. This study represents the first report of the occurrence and mixed infections of *E. bieneusi*, *E. cuniculi*, *E. hellem, Cryptosporidium*, and *G. duodenalis* in diarrheic pigs in Chongqing and Sichuan Provinces. The prevalence of *E. bieneusi*, *E. cuniculi*, *E. hellem, Cryptosporidium*, and *G. duodenalis* was 79.8% (410/514) [29], 12% (62/514), 4% (21/514), 0% (0/514) and 8.95% (46/514) among fecal samples, respectively. The present study results show that enteric pathogens are common in diarrheic pigs in Chongqing and Sichuan Provinces. On the 14 farms studied, fecal samples from diarrheic pigs harbored at least one enteric pathogen. *E. bieneusi, E. cuniculi, E. hellem* and *G. duodenalis* assemblage A were the common pathogens causing diarrhea in pigs.

*E. bieneusi*, *E. intestinalis*, *E. hellem*, and *E. cuniculi* are the most common causes of microsporidiosis in humans [14, 42]. The overall prevalence of *E. cuniculi* and *E. hellem* were 12% (62/514) and 4% (21/514), respectively. However, no infection of *E. intestinalis* was detected in the diarrheic pigs in our study. The prevalence of *E. cuniculi* detected in our study is higher than that reported by Nemeji et al.[43] 3.5% (16/460), in wild boards from Central Europe and by Reetz et al. [44] 8.8% (3/34) in domestic pigs from Germany. A recent study performed by Pekmezci et al. [38] on budgerigar from Turkey revealed a 14.7% (21/143) prevalence of *E. hellem*, much higher than that detected in our study.

In this study, the sequences of amplicons from *E. cuniculi*-positive samples were determined to belong to Genotypes II, and III, with Genotype III showing dominance (77.4%, 48/62) among the detected genotypes. A study on wild boars in Central Europe revealed a higher prevalence of *E. cuniculi* genotype II (61.9%, 13/21) [43]. Regarding the *Encephalitozoon* spp. infection of pigs, this study is the first report from Southwestern China, and a similar genotype of *E. hellem* (TURK1B) was identified in budgerigar from Turkey [38], but there are numerous reports of *E. hellem* in various bird hosts and mammals [45]. To the researchers’ knowledge, this is the first report of the TURK1B genotype of *E. hellem* in pigs, and further study is needed to investigate the relationship between budgerigars and pigs.

Different *Cryptosporidium* species cause cryptosporidiosis in pigs. At least 42 species and 70 genotypes of *Cryptosporidium* spp. have been found in different hosts. Twenty *Cryptosporidium* species have been found in humans and eight in pigs. Six of the eight *Cryptosporidium* species found in pigs [*C. scrofarum*, *C. suis*, *C. parvum*, *C. muris*, *C. andersoni*, and *C. tyzzeri*] can infect pigs naturally. In contrast, the other two species [*C. hominis* and *C. meleagridis*] can infect pigs only by human intervention [41]. In addition, infections with *Cryptosporidium* have been identified in pigs of all age groups in multiple developed and developing countries [46, 47]. The present study detected no *Cryptosporidium* species-positive samples upon amplification of the SSU rRNA genes using nested PCR. Our results were similar to that in previous reports from Hunan [41], Ezhou, Xiaogan, Xiangyang [48], Zhengzhou (farms 2,3 and 4), Zhoukou and Luohe [49]. The lack of detection may be attributed to the well-managed breeding practices of farms [41]. Furthermore, previous studies indicated that *Cryptosporidium* spp. were associated with asymptomatic pigs, *Cryptosporidium* spp. might not be a direct cause of diarrhea in pigs [50, 51], while it is associated with diarrhea in alpacas and calves [52, 53]. However, a recent meta-analysis study by Wang and colleagues showed a 10.5% and 8.2% prevalence in pigs in Chongqing and Sichuan Provinces, respectively [54]. In addition, different prevalences of *Cryptosporidium* spp. in pigs have been reported in different provinces of China, with the highest in Heilongjiang Province (55.8%, 63/113) [55] and the lowest in the Tibet Autonomous Region (0.49%, 3/614) [56].

Based on the β-giardin (bg) genes of *G. duodenalis*, an overall prevalence of 8.95% (46/514) was detected in diarrheic pigs in Southwest China. The infection rates of *G. duodenalis* in pigs were lower than those previously reported in other provinces of China, i.e., Shanghai (26.88%, 25/93) [57] and Zhejiang (10.5%, 13/124) [58], and other parts of the world, i.e., in western Australia (31.1%, 90/289) [59], northwestern of England (57.1%, 4/7) [60], and Ontario Canada (66.4%, 81/122) [61]. However, they were higher than those reported in Hubei (0.97%, 8/826), Xinjiang (2.6%, 21/801), Henan (1.7%, 15/897), Yunnan (2.5%, 5/200), and Guangdong (4.2%, 3/72) [23, 49, 58, 62]. Many factors can affect the prevalence of *G. duodenalis* in pigs, such as the age groups, methods of breeding, management system, detection methods, water supply, farm hygiene, animal stocking density, and pig health status [49, 57].

*Giardia duodenalis* infections occur in all age groups of pigs, from nursing piglets to adult sows [63]. In this study, *G. duodenalis* had the highest prevalence in suckling pigs (10.8%, 39/326) and the lowest in weaned pigs (0%, 0/17). Our rates were lower than those revealed in a study in Australia (18.7%, 23/123) [59] but higher than those in Denmark (2%, 3/152) [64], Zambia (6.3%, 2/32) [65], southern China (2.3%, 2/87) [58], and Shaanxi province (6.5%, 10/155) [66]. More studies from Xinjiang and three Provinces of southern China (Yunnan, Zhejiang and Guangdong) found a higher prevalence in fattening pigs (5.4%, 7/129) and (12.3, 9/73), respectively [58, 62]. In contrast, the highest *G. duodenalis* prevalence was found in weaned pigs in Denmark (27.4%, 64/234) [64] and Australia (41.0%, 64/156) [59]. Moreover, other studies detected a higher prevalence in sows in Hubei Province of China (1.38%, 5/362) [23], Shaanxi Province of China (10.5%, 6/57) [66], and Zambia (40.0%, 6/15) [65]. Such variation may be due differences into sample size.

A previous review [67] showed that *G. duodenalis* assemblage A is reported to infect domestic animals, wild animals (including beavers, cats, lemurs, cows, sheep, dogs, chinchillas, alpacas, horses, and pigs), and humans. In this study, analysis of the DNA sequence of the β-giardin (bg) gene showed that all isolates belonged to *G. duodenalis* assemblage A and subassemblage A1, consistent with previous reports in humans and calves [32]. However, a higher prevalence of *G. duodenalis* assemblage E was reported in Shaanxi Province (80%, 36/45) [66], Henan Province (60%, 9/15) [49], Hubei Province (87.5%, 7/8), Denmark (84.6%, 11/67) [64] and Australia (12.8%, 37/289) [59]. The higher prevalence of *G. duodenalis* assemblage A in Chongqing and Sichuan Provinces is worthy of further study. The present results, in line with those of previous reports, suggest that pigs are a potential source of environmental contamination and infection for humans.

Out of the 514 fecal samples, considering all five enteric pathogens assessed in this study, 322 diarrheic pigs had mono-infections, accounting for 62.6% of the overall prevalence. In all of these cases, *E. bieneusi*, *E. cuniculi*, *E. hellem* and *G. duodenalis* were present. However, no infection with *E. intestinalis* or *Cryptosporidium* species was detected in this study. In addition to single infections, we found mixed infections in 106 samples, which accounted for 20.6% of the total positive samples. *E. bieneusi* was found more often than the other enteric pathogens, which is consistent with the results of a study performed on wild boar [43], pet rabbits [22], and horses [68]. The cause for multiple infections may be contaminated sewage. To the best of our knowledge, this is the first report of coinfection of *E. bieneusi* with other enteric pathogens in China. Therefore, further investigation should be conducted to confirm whether *E. bieneusi* is the main cause of diarrhea in pigs.

## Conclusions

This study detected the occurrence of mono-infection and concurrent infection of *E. bieneusi, Encephalitozoon* spp., *Cryptosporidium* spp. and *G. duodenalis* in diarrheic pigs in China. Mono-infections and coinfections of *E. bieneusi, Encephalitozoon* spp., and *G. duodenalis* were detected in 62.6% and 20.6% of fecal samples, respectively. No infections of *E. intestinalis* or *Cryptosporidium* species were detected in any of our samples. The highest rate of coinfection was detected between *E. bieneusi* and *E. cuniculi* (10.5%, 54/514), followed by *E. bieneusi* and *G. duodenalis* (5.8%, 30/514) and *E. bieneusi* and *E. hellem* (2.9%, 15/514). *E. bieneusi* is a prevalent pathogen in diarrheic pigs and may be a significant source of diarrheal disease. Therefore, farmers, veterinary workers, health workers, and people who have close contact with pigs should take care to avoid being infected by these enteric pathogens. Moreover, our data showed that enteric pathogens were present and likely common in diarrheic pigs in Chongqing and Sichuan Provinces and could be a potential source for zoonotic transmission in humans and other animals and environmental contamination. Further investigation is needed on farms with diverse breeding systems and animals of various ages and both sexes to determine whether *E. bieneusi* is the main cause of diarrhea in pigs. In addition, a long-term study, including the viruses and bacterial infecting our samples, is needed.

### Electronic supplementary material

Below is the link to the electronic supplementary material.

Supplementary Material 1

## Data Availability

The datasets generated for this study can be found in GenBank under the accession numbers OR058746 to OR058757 for *Encephalitozoon* spp. and OR091265 to OR091271 *Giardia duodenalis*.

## References

[1] Theuns S, Vyt P, Desmarets LMB, Roukaerts IDM, Heylen E, Zeller M (2016). Presence and characterization of pig group A and C rotaviruses in feces of Belgian diarrheic suckling piglets. Virus Res.

[2] Ruiz VL, Bersano JG, Carvalho AF, Catroxo MH, Chiebao DP, Gregori F (2016). Case-control study of pathogens involved in piglet diarrhea. BMC Res Notes.

[3] Li Y, Qiu X, Li H, Zhang Q (2007). Adhesive patterns of *Escherichia coli* F4 in piglets of three breeds. J Genet Genomics.

[4] Carter HSM, Renaud DL, Steele MA, Fischer-Tlustos AJ, Costa JHC. A narrative review on the unexplored potential of Colostrum as a Preventative Treatment and Therapy for Diarrhea in neonatal dairy calves. Anim (Basel). 2021;11(8). 10.3390/ani11082221. 10.3390/ani11082221 PMC8388388 34438679

[5] Qiu L, Xia W, Li W, Ping J, Ding S, Liu H (2019). The prevalence of microsporidia in China: a systematic review and meta-analysis. Sci Rep.

[6] Zhang K, Zheng S, Wang Y, Wang K, Wang Y, Gazizova A (2021). Occurrence and molecular characterization of *Cryptosporidium* spp., *Giardia duodenalis*, *Enterocytozoon bieneusi*, and *Blastocystis* spp. in captive wild animals in zoos in Henan, China. BMC Vet Res.

[7] Taghipour A, Bahadory S, Khazaei S, Zaki L, Ghaderinezhad S, Sherafati J (2022). Global molecular epidemiology of microsporidia in pigs and wild boars with emphasis on *Enterocytozoon bieneus*i: a systematic review and meta-analysis. Vet Med Sci.

[8] Pettersson E, Ahola H, Frössling J, Wallgren P, Troell K (2020). Detection and molecular characterisation of *Cryptosporidium* spp. in Swedish pigs. Acta Vet Scand.

[9] Stentiford G, Becnel J, Weiss L, Keeling P, Didier E, Bjornson S (2016). Microsporidia–emergent pathogens in the global food chain. Trends Parasitol.

[10] Valenčáková A, Sučik M. Alternatives in molecular diagnostics of encephalitozoon and enterocytozoon infections. J Fungi (Basel). 2020;6(3). 10.3390/jof6030114. 10.3390/jof6030114 PMC7558530 32707956

[11] Mena CJ, Barnes A, Castro G, Guasconi L, Burstein VL, Beccacece I (2021). Microscopic and PCR-based detection of microsporidia spores in human stool samples. Rev Argent Microbiol.

[12] Han B, Pan G, Weiss LM (2021). Microsporidiosis in Humans Clin Microbiol Rev.

[13] Han B, Weiss LM (2017). Microsporidia: obligate intracellular pathogens within the fungal kingdom. Microbiol Spectr.

[14] Li W, Feng Y, Santin M (2019). Host Specificity of *Enterocytozoon bieneusi* and Public Health implications. Trends Parasitol.

[15] Li W, Feng YY, Zhang LX, Xiao LH (2019). Potential impacts of host specificity on zoonotic or interspecies transmission of *Enterocytozoon bieneusi*. Infect Genet Evol.

[16] Magalhães TR, Pinto FF, Queiroga FL (2022). A multidisciplinary review about *Encephalitozoon cuniculi* in a one health perspective. Parasitol Res.

[17] Kahler AM, Thurston-Enriquez JA (2007). Human pathogenic microsporidia detection in agricultural samples: method development and assessment. Parasitol Res.

[18] Didier ES, Vossbrinck CR, Baker MD, Rogers LB, Bertucci DC, Shadduck JA (1995). Identification and characterization of three *Encephalitozoon cuniculi* strains. Parasitology.

[19] Talabani H, Sarfati C, Pillebout E, van Gool T, Derouin F, Menotti J (2010). Disseminated Infection with a new genovar of *Encephalitozoon cuniculi* in a renal transplant recipient. J Clin Microbiol.

[20] Mathis A, Weber R, Deplazes P (2005). Zoonotic potential of the microsporidia. Clin Microbiol Rev.

[21] Mathis A, Tanner I, Weber R, Deplazes P (1999). Genetic and phenotypic intraspecific variation in the microsporidian *Encephalitozoon hellem*. Int J Parasitol.

[22] Deng L, Chai Y, Xiang L, Wang W, Zhou Z, Liu H (2020). First identification and genotyping of *Enterocytozoon bieneusi* and *Encephalitozoon* spp. in pet rabbits in China. BMC Vet Res.

[23] Li D, Deng H, Zheng Y, Zhang H, Wang S, He L (2022). First characterization and zoonotic potential of *Cryptosporidium* spp. and *Giardia duodenalis* in pigs in Hubei Province of China. Front Cell Infect Microbiol.

[24] Ryan UM, Feng Y, Fayer R, Xiao L (2021). Taxonomy and molecular epidemiology of *Cryptosporidium* and *Giardia *- a 50 year perspective (1971–2021). Int J Parasitol.

[25] Xu J, Liu H, Jiang Y, Jing H, Cao J, Yin J (2022). Genotyping and subtyping of *Cryptosporidium* spp. and *Giardia duodenalis* isolates from two wild rodent species in Gansu Province, China. Sci Rep.

[26] Dong S, Yang Y, Wang Y, Yang D, Yang Y, Shi Y (2020). Prevalence of *Cryptosporidium* Infection in the Global Population: a systematic review and Meta-analysis. Acta Parasitol.

[27] Feng Y, Xiao L (2011). Zoonotic potential and molecular epidemiology of Giardia species and giardiasis. Clin Microbiol Rev.

[28] Xiao L, Feng Y. Molecular epidemiologic tools for waterborne pathogens cryptosporidium spp. and giardia duodenalis. Food Waterborne Parasitol. 2017;8–9. 10.1016/j.fawpar.2017.09.002. 10.1016/j.fawpar.2017.09.002 PMC7034008 32095639

[29] Ghebremichael ST, Meng X, Wei J, Yang Y, Huang Q, Luo L (2022). Prevalence and genotyping distribution of *Enterocytozoon bieneusi* in diarrheic pigs in Chongqing and Sichuan Provinces, China. Front Microbiol.

[30] Katzwinkel-Wladarsch S, Lieb M, Helse W, Löscher T, Rinder H (1996). Direct amplification and species determination of microsporidian DNA from stool specimens. Trop Med Int Health.

[31] Franzen C, Müller A (1999). Molecular techniques for detection, species differentiation, and phylogenetic analysis of microsporidia. Clin Microbiol Rev.

[32] Lalle M, Pozio E, Capelli G, Bruschi F, Crotti D, Cacciò SM. Genetic heterogeneity at the beta-giardin locus among human and animal isolates of giardia duodenalis and identification of potentially zoonotic subgenotypes Int J Parasitol, 2005. 35(2): p. 207 – 13 10.1016/j.ijpara.2004.10.022. 10.1016/j.ijpara.2004.10.022 15710441

[33] Xiao L, Escalante L, Yang C, Sulaiman I, Escalante AA, Montali RJ (1999). Phylogenetic analysis of *Cryptosporidium* parasites based on the small-subunit rRNA gene locus. Appl Environ Microbiol.

[34] Xiao L, Singh A, Limor J, Graczyk TK, Gradus S, Lal A (2001). Molecular characterization of *Cryptosporidium* oocysts in samples of raw surface water and wastewater. Appl Environ Microbiol.

[35] Buckholt MA, Lee JH, Tzipori S (2002). Prevalence of *Enterocytozoon bieneusi* in swine: an 18-month survey at a slaughterhouse in Massachusetts. Appl Environ Microbiol.

[36] Perec-Matysiak A, Leśniańska K, Buńkowska-Gawlik K, Čondlová Å, Sak B, Kváč M (2019). The opportunistic pathogen *Encephalitozoon cuniculi* in wild living Murinae and Arvicolinae in Central Europe. Eur J Protistol.

[37] Hofmannová L, Sak B, Jekl V, Mináriková A, Skorič M, Kváč M (2014). Lethal *Encephalitozoon cuniculi* genotype III Infection in steppe lemmings (*Lagurus lagurus*). Vet Parasitol.

[38] Pekmezci D, Yetismis G, Esin C, Duzlu O, Colak ZN, Inci A (2020). Occurrence and molecular identification of zoonotic microsporidia in pet budgerigars (*Melopsittacus undulatus*) in Turkey. Med Mycol.

[39] Prystajecky N, Tsui CK, Hsiao WW, Uyaguari-Diaz MI, Ho J, Tang P (2015). *Giardia* spp. Are commonly found in mixed assemblages in Surface Water, as revealed by Molecular and whole-genome characterization. Appl Environ Microbiol.

[40] Ye J, Xiao L, Wang Y, Guo Y, Roellig DM, Feng Y (2015). Dominance of *Giardia duodenalis* assemblage A and *Enterocytozoon bieneusi* genotype BEB6 in sheep in Inner Mongolia, China. Vet Parasitol.

[41] Wang P, Li S, Zou Y, Du ZC, Song DP, Wang P (2022). The Infection and molecular characterization of *Cryptosporidium* spp. in diarrheic pigs in southern China. Microb Pathog.

[42] Ryan ET, Hill DR, Solomon T, Aronson N, Endy TP. Hunter’s tropical medicine and emerging infectious diseases e-book. Elsevier Health Sciences; 2019.

[43] Němejc K, Sak B, Květoňová D, Hanzal V, Janiszewski P, Forejtek P (2014). Prevalence and diversity of *Encephalitozoon * spp. and * Enterocytozoon bieneusi * in wild boars (*Sus scrofa*) in Central Europe. Parasitol Res.

[44] Reetz J, Nöckler K, Reckinger S, Vargas MM, Weiske W, Broglia A (2009). Identification of *Encephalitozoon cuniculi* genotype III and two novel genotypes of *Enterocytozoon bieneusi* in swine. Parasitol Int.

[45] Hinney B, Sak B, Joachim A, Kváč M (2016). More than a rabbit’s tale - *Encephalitozoon* spp. in wild mammals and birds. Int J Parasitol Parasites Wildl.

[46] De Felice LA, Moré G, Cappuccio J, Venturini MC, Unzaga JM (2020). Molecular characterization of *Cryptosporidium* spp. from domestic pigs in Argentina. Vet Parasitol Reg Stud Reports.

[47] Qi M, Zhang Q, Xu C, Zhang Y, Xing J, Tao D (2020). Prevalence and molecular characterization of *Cryptosporidium* spp. in pigs in Xinjiang, China. Acta Trop.

[48] Seatamanoch N, Kongdachalert S, Sunantaraporn S, Siriyasatien P, Brownell N. Microsporidia, a highly adaptive organism and its host expansion to humans. Front Cell Infect Microbiol, 2022: p. 809. 10.3389/fcimb.2022.924007 PMC9245026 35782144

[49] Wang H, Zhang Y, Wu Y, Li J, Qi M, Li T (2018). Occurrence, molecular characterization, and Assessment of zoonotic risk of *Cryptosporidium* spp., *Giardia duodenalis*, and *Enterocytozoon bieneusi* in pigs in Henan, Central China. J Eukaryot Microbiol.

[50] Nguyen ST, Honma H, Geurden T, Ikarash M, Fukuda Y, Huynh VV (2012). Prevalence and risk factors associated with *Cryptosporidium* oocysts shedding in pigs in Central Vietnam. Res Vet Sci.

[51] Nguyen ST, Fukuda Y, Tada C, Sato R, Huynh VV, Nguyen DT (2013). Molecular characterization of *Cryptosporidium* in pigs in central Vietnam. Parasitol Res.

[52] Gomez-Puerta LA, Gonzalez AE, Vargas-Calla A, Lopez-Urbina MT, Cama V, Xiao L (2020). *Cryptosporidium parvum* as a risk factor of diarrhea occurrence in neonatal alpacas in Peru. Parasitol Res.

[53] Foster DM, Smith GW. Pathophysiology of diarrhea in calves. Vet Clin North Am Food Anim Pract, 2009. 25(1): p. 13–36, xi 10.1016/j.cvfa.2008.10.013. 10.1016/j.cvfa.2008.10.013 PMC7125768 19174281

[54] Wang W, Gong QL, Zeng A, Li MH, Zhao Q, Ni HB (2021). Prevalence of *Cryptosporidium* in pigs in China: a systematic review and meta-analysis. Transbound Emerg Dis.

[55] Zhang W, Yang F, Liu A, Wang R, Zhang L, Shen Y (2013). Prevalence and genetic characterizations of *Cryptosporidium* spp. in pre-weaned and post-weaned piglets in Heilongjiang Province, China. PLoS ONE.

[56] Zheng S, Li D, Zhou C, Zhang S, Wu Y, Chang Y (2019). Molecular identification and epidemiological comparison of *Cryptosporidium* spp. among different pig breeds in Tibet and Henan, China. BMC Vet Res.

[57] Liu H, Xu N, Yin J, Yuan Z, Shen Y, Cao J (2019). Prevalence and multilocus genotyping of potentially zoonotic *Giardia duodenalis* in pigs in Shanghai, China. Parasitology.

[58] Zou Y, Yuan XD, Zhang SY, Zhang HY, Chen XQ. Molecular detection and characterization of giardia duodenalis in Farmed pigs in three Provinces of Southern China. Pathogens. 2021;10(11). 10.3390/pathogens10111481. 10.3390/pathogens10111481 PMC8625397 34832636

[59] Armson A, Yang R, Thompson J, Johnson J, Reid S, Ryan UM (2009). *Giardia* genotypes in pigs in Western Australia: prevalence and association with diarrhea. Exp Parasitol.

[60] Minetti C, Taweenan W, Hogg R, Featherstone C, Randle N, Latham SM (2014). Occurrence and diversity of *Giardia duodenalis* assemblages in livestock in the UK. Transbound Emerg Dis.

[61] Farzan A, Parrington L, Coklin T, Cook A, Pintar K, Pollari F (2011). Detection and characterization of *Giardia duodenalis* and cryptosporidium spp. on swine farms in Ontario, Canada. Foodborne Pathog Dis.

[62] Jing B, Zhang Y, Xu C, Li D, Xing J, Tao D (2019). Detection and genetic characterization of *Giardia duodenalis* in pigs from large-scale farms in Xinjiang, China. Parasite.

[63] Maddox-Hyttel C, Langkjaer RB, Enemark HL, Vigre H (2006). *Cryptosporidium* and *Giardia* in different age groups of Danish cattle and pigs–occurrence and management associated risk factors. Vet Parasitol.

[64] Petersen HH, Jianmin W, Katakam KK, Mejer H, Thamsborg SM, Dalsgaard A (2015). *Cryptosporidium* and *Giardia* in Danish organic pig farms: Seasonal and age-related variation in prevalence, Infection intensity and species/genotypes. Vet Parasitol.

[65] Siwila J, Mwape KE (2012). Prevalence of *Cryptosporidium* spp. and *Giardia duodenalis* in pigs in Lusaka, Zambia. Onderstepoort J Vet Res.

[66] Wang SS, Yuan YJ, Yin YL, Hu RS, Song JK, Zhao GH (2017). Prevalence and multilocus genotyping of *Giardia duodenalis* in pigs of Shaanxi Province, northwestern China. Parasit Vectors.

[67] Adam RD (2001). Biology of *Giardia lamblia*. Clin Microbiol Rev.

[68] Wagnerová P, Sak B, Květoňová D, Buňatová Z, Civišová H, Maršálek M (2012). *Enterocytozoon bieneusi* and *Encephalitozoon cuniculi *in horses kept under different management systems in the Czech Republic. Vet Parasitol.

