# Supplementary material for: First identification and coinfection detection of Enterocytozoon bieneusi, Encephalitozoon spp., Cryptosporidium spp. and Giardia duodenalis in diarrheic pigs in Southwest China
Source: BMC Microbiol. 2023 Nov 11;23:334. doi: 10.1186/s12866-023-03070-x (PMC10640745; doi:10.1186/s12866-023-03070-x)

Supplementary Material

## Supplementary Figures

**Supplementary Figure S1 (A).** Multiple alignment of *E. cuniculi* Genotype II


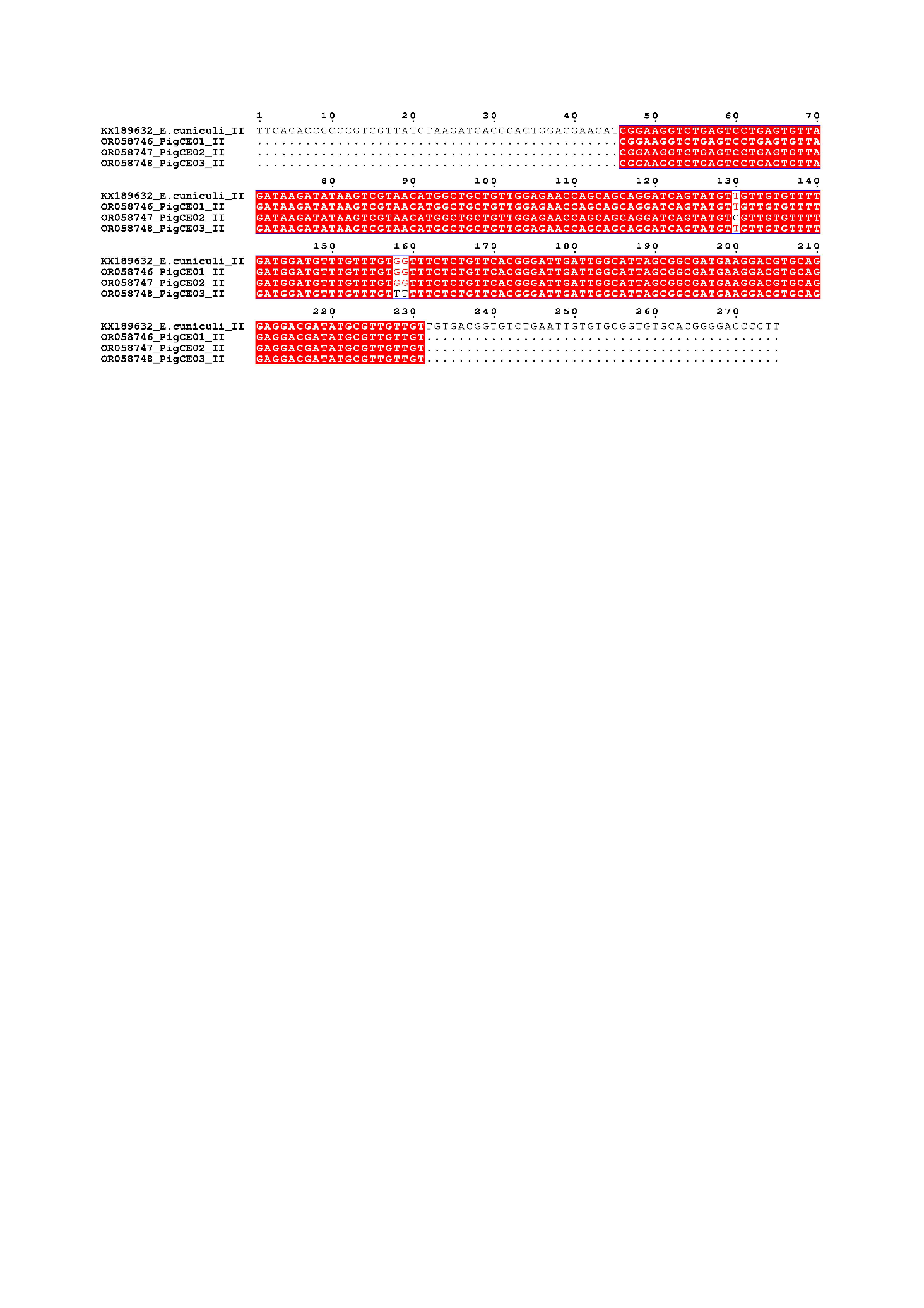


**Supplementary Figure 1** (B). Multiple Alignment of *E. cuniculi* Genotype III


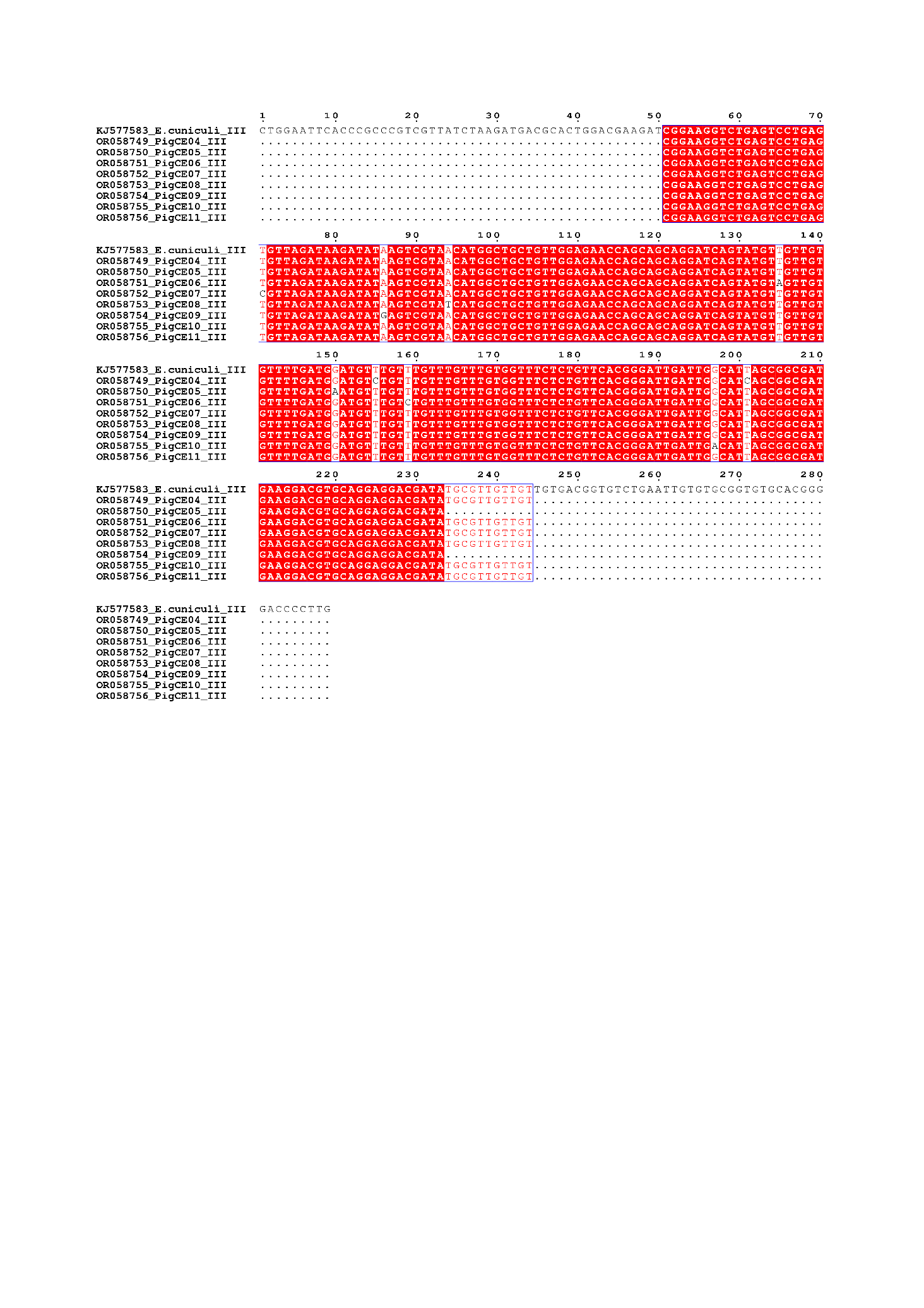


**Supplementary Figure 1** **(C).** Multiple *E. hellem* **Genotype**
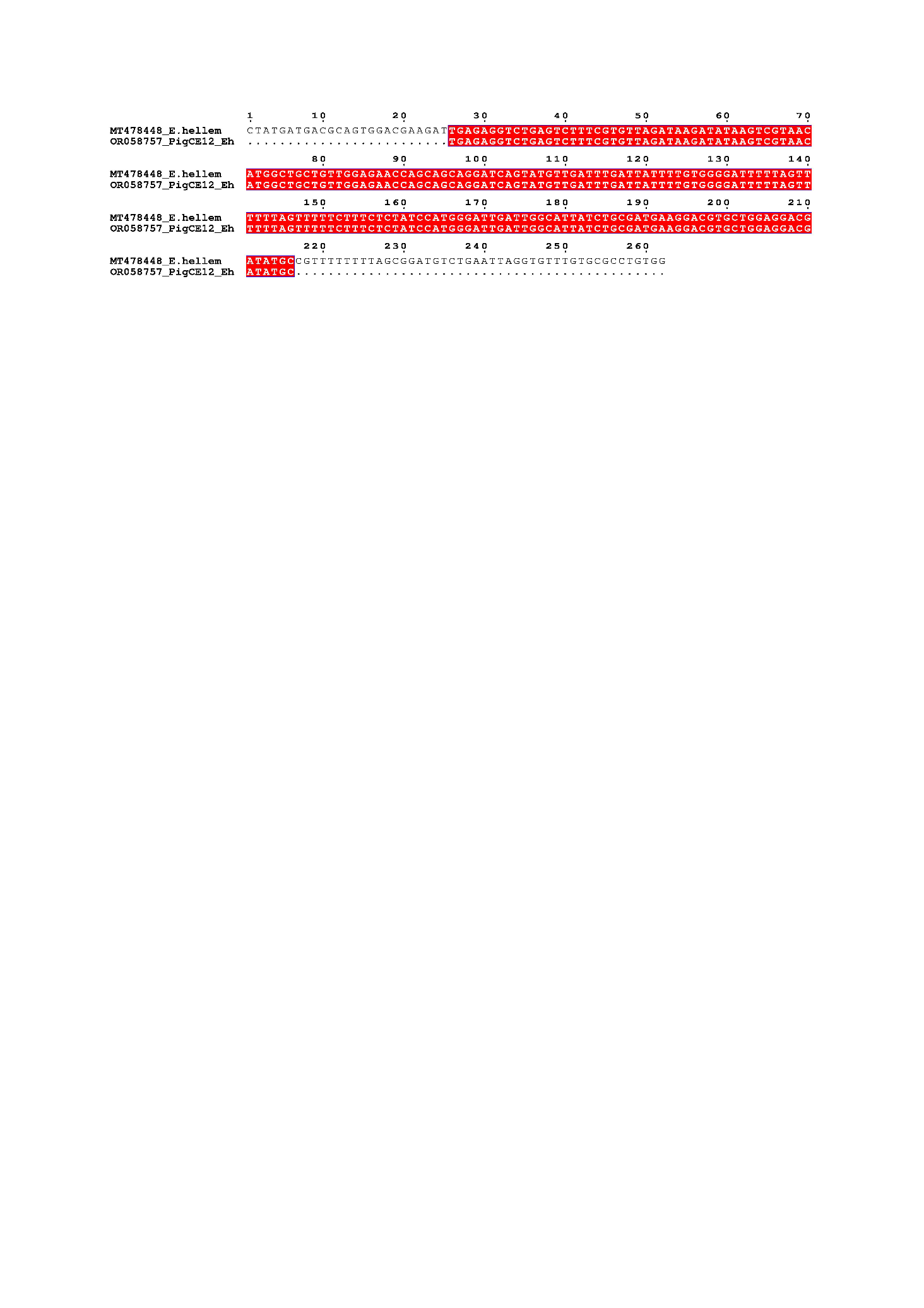


**Supplementary Figure 1(D).** Multiple alignment of *G. duodenalis* assemblage A


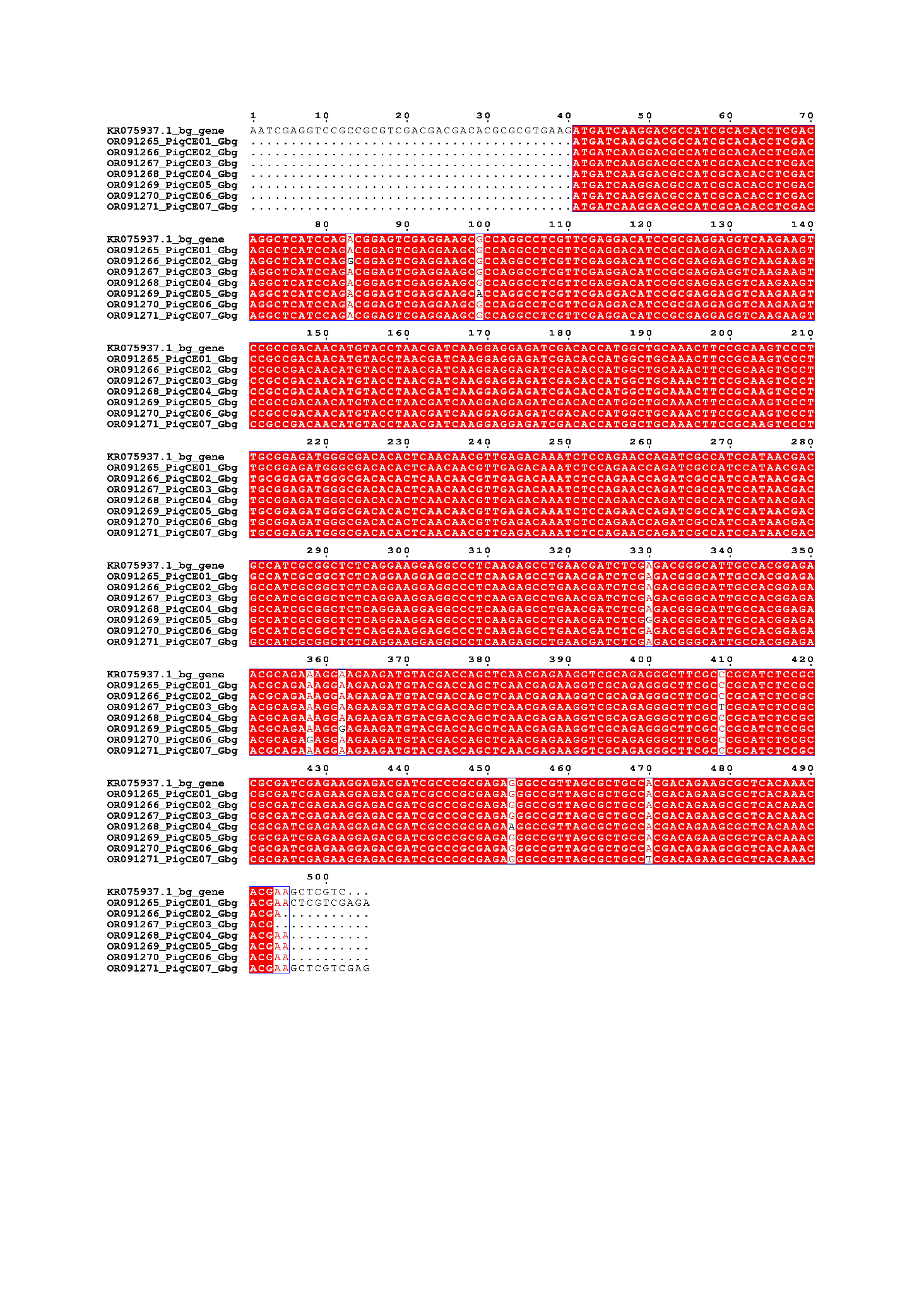


**Supplementary Figure 2.** Sampling map coinfection of *Enterocytozoon bieneusi* to other enteric pathogens in Chongqing and Sichuan Provinces, China (<https://d-maps.com/carte.php?num_car=27749&lang=zh>)


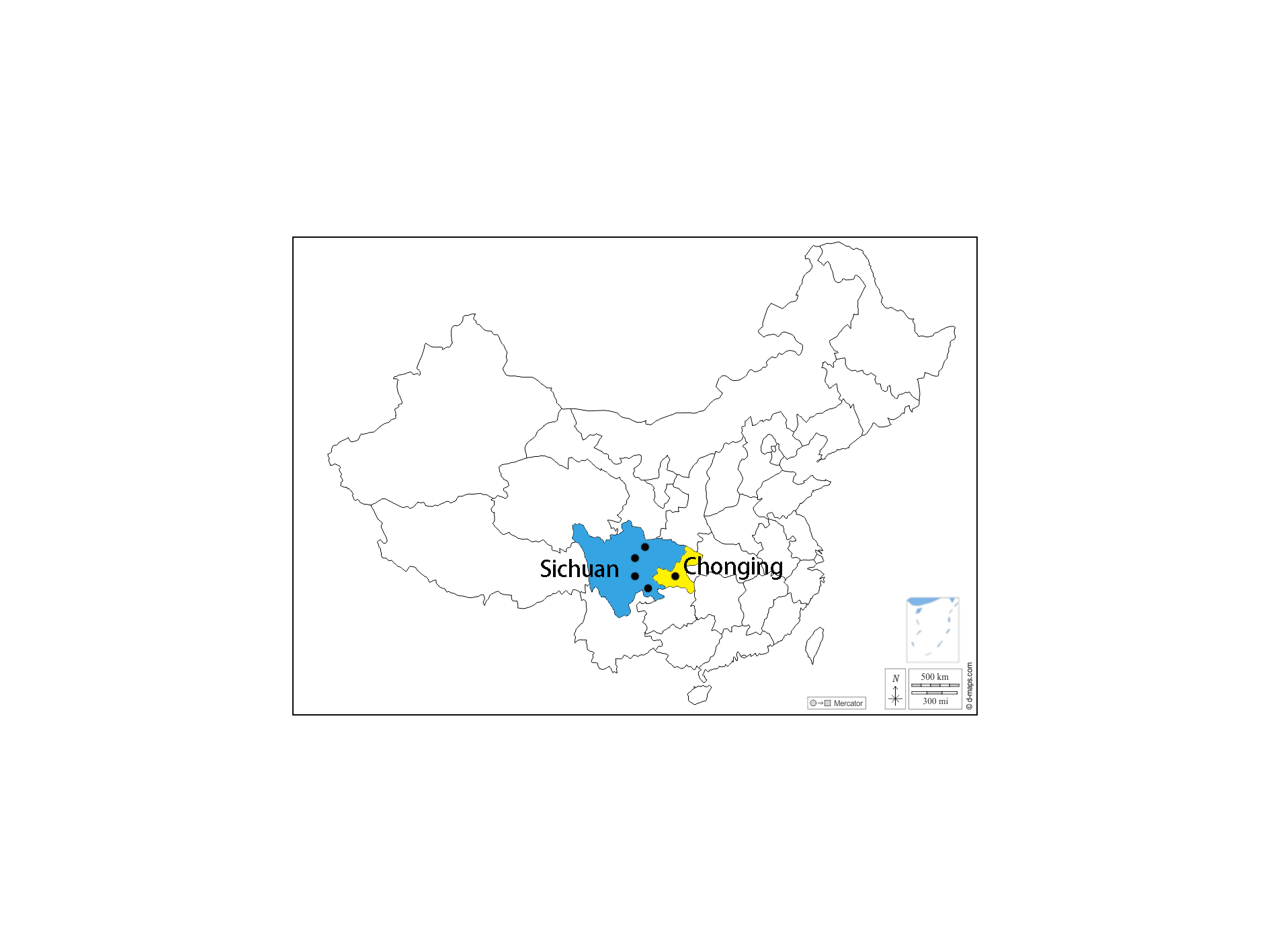

Supplement: Supplementary file 1 — Supplementary Material 1 [file 12866_2023_3070_MOESM1_ESM.docx]
